# Vitamin D Deficiency as a Predictor of a High Prevalence of Coronary Artery Disease in Pancreas Transplant Candidates With Type 1 Diabetes

**DOI:** 10.3389/fendo.2021.714728

**Published:** 2021-08-11

**Authors:** Małgorzata Buksińska-Lisik, Przemysław J. Kwasiborski, Robert Ryczek, Wojciech Lisik, Artur Mamcarz

**Affiliations:** ^1^3rd Department of Internal Medicine and Cardiology, Medical University of Warsaw, Warsaw, Poland; ^2^Department of Cardiology and Internal Diseases, Military Institute of Medicine, Warsaw, Poland; ^3^Department of General and Transplantation Surgery, The Medical University of Warsaw, Warsaw, Poland

**Keywords:** pancreas transplantation, coronary artery disease, cardiovascular risk, vitamin D, 25-hydroxyvitamin D, type 1 diabetes

## Abstract

**Introduction:**

Pancreas transplantation is a high-risk procedure in terms of cardiovascular complications. Therefore, identification of all cardiovascular risk factors is crucial to prevent cardiovascular complications after pancreas transplantation. Vitamin D deficiency (VDD) appears to be a potential risk factor for coronary artery disease.

**Objective:**

To determine the prevalence of VDD in pancreas transplant candidates, and further to examine the relationship between vitamin D and the prevalence of coronary artery disease and lipid profile parameters.

**Materials and Methods:**

This is a prospective cross-sectional study. We enrolled consecutive patients with type 1 diabetes eligible for simultaneous pancreas-kidney transplantation or pancreas transplant alone. The laboratory tests included HbA1c, lipid profile, creatinine, and total 25-hydroxyvitamin D (25(OH)D). The diagnosis of coronary artery disease was based on coronary angiography.

**Results:**

The study population included 48 patients. VDD was revealed in 48% of patients and coronary artery disease in 35% of patients. The mean concentration of vitamin D in the entire cohort was 21.3 ± 9.48 ng/ml. The median value of 25(OH)D in patients with coronary artery disease was significantly lower than in patients without coronary artery disease (18.5 (11.6–21.5) *vs*. 24.8 (18.4–31.8) ng/ml, p = 0.018). There was a significant relationship between VDD and coronary artery disease (OR = 4.36; 95% confidence interval (CI): 1.22–15.64, p = 0.034). A patient’s odds of having coronary artery disease while having a sufficient level of vitamin D was 4.36 times lower than if the patient had VDD. There was a significant relationship between VDD and hypertension (OR = 5.91; 95% CI: 1.12–31.20, p = 0.039) and hemodialysis (OR = 4.25; 95% CI: 1.25–14.5, p = 0.023). There was no significant correlation between 25(OH)D and lipid profile.

**Conclusions:**

VDD is highly prevalent in pancreas transplant candidates with type 1 diabetes. There is a significant relationship between VDD and increased prevalence of coronary disease. The lack of any significant association between serum vitamin D and lipid profile suggests that the relationship between vitamin D and coronary artery disease results from other causes.

## Introduction

Pancreas transplantation is an established method of treatment in selected patients with type 1 diabetes (T1D). There are two main methods of pancreas transplantation: pancreas transplant alone (PTA) and simultaneous pancreas kidney transplantation (SPKT), the latter being a preferred treatment option for patients with end-stage diabetic kidney disease. Both procedures allow for sustained glycemic control and improve prognosis in T1D patients (1, 2). Nevertheless, pancreas transplantation is associated with a high risk of cardiovascular events and cardiovascular complications are still the most common cause of death in this group of patients (3, 4).

T1D patients are at increased risk of cardiovascular disease (CVD) due to both the adverse effects of hyperglycemia and the coexistence of other additional risk factors, including hypertension and dyslipidemia (5). In addition to the main CVD risk factors, additional, non-classical risk factors are thought to affect the prevalence of coronary artery disease (CAD). The deficiency of vitamin D is one of such postulated risk factors (6).

Vitamin D deficiency (VDD) is a worldwide problem. It is estimated to affect about 40% of the European population (7). In Poland, VDD was found in 65.8% of adults (<20 ng/ml), while the optimal level (>30–50 ng/ml) in only 9.1% (8). People with T1D present VDD more often than the general population (9). Among Polish adolescents diagnosed with T1D, VDD was found in 82% (compared to 67% of young individuals without diabetes) (10). Low vitamin D levels are associated with the risk of coronary events and cardiovascular death (11, 12). Observational studies conducted in the general population suggest that a potential factor linking VDD with CAD may be an adverse effect on the lipid profile (13, 14). However, studies aimed to assess the impact of VDD on the lipid profile in patients with T1D concern mainly children and the results of the analyses are inconclusive (15, 16). To the best of our knowledge, studies evaluating the association between VDD and the prevalence of CAD and lipid profile in patients with long-standing T1D eligible for pancreas transplantation have not been conducted yet.

Therefore, this study aims to determine the prevalence of VDD in pancreas transplant candidates with T1D, and to examine the relationship between vitamin D status and the prevalence of CAD and between vitamin D and lipid profile.

## Materials and Methods

### Study Population

This cross-sectional study was conducted at the 3rd Department of Internal Medicine and Cardiology, Medical University of Warsaw (Poland). All consecutive patients with T1D eligible for SPKT or PTA who were referred to our center for cardiological pretransplant assessment were prospectively enrolled in the study. Patients were recruited from August 2018 to September 2020. The study protocol was approved by the local Bioethics Committee of the Medical University of Warsaw (Poland). All participants signed an informed consent form to participate in the study after a comprehensive explanation of the study details.

### Anthropometric and Laboratory Measurements

The following demographic and medical data were collected: age, sex, type of planned transplantation procedure, age at onset and duration of T1D, renal replacement therapy, previous cardiovascular disease (CAD, stroke, peripheral artery disease), and the main risk factors for CVD (hypertension, smoking habit, dyslipidemia). Hypertension was defined as a systolic blood pressure (SBP) >140 mmHg or diastolic blood pressure (DBP) >90 mmHg and/or by the use of antihypertensive therapy prior to enrollment into the study. Dyslipidemia was defined when total cholesterol (TC) was ≥5.2 mmol/L and/or low-density lipoprotein-cholesterol (LDL-C) was ≥3.0 mmol/L and/or triglycerides (TG) were ≥1.7 mmol/L or a patient was on lipid-lowering therapy. All the patients were rated in relation to the drug treatment for dyslipidemia and vitamin D supplementation.

Height (m) and weight (kg) were measured before breakfast with light clothes. The weight of people on hemodialysis (HD) was measured on a non-dialysis day. BMI was calculated as weight in kilograms divided by height in meters squared. Underweight was defined as a BMI under 18.5 kg/m^2^, normal weight from 18.5 to 24.9, overweight from 25 to 29.9, and obesity as a BMI ≥ 30 kg/m^2^.

Venous blood was collected in the fasting state. Concentrations of total cholesterol (TC), high-density lipoprotein-cholesterol (HDL-C), TG, glycated hemoglobin (HbA1c), and creatinine (Cr) in plasma were measured using a Beckman Coulter AU analyzer (Beckman Coulter, Inc. Brea, CA, USA). The concentration of low-density lipoprotein-cholesterol (LDL-C) was calculated using the Friedewald formula (17). Non-high-density lipoprotein-cholesterol (non-HDL-C) was calculated as non-HDL-C = TC - HDL-C. Glomerular filtration rate (eGFR) was estimated by using the Modification of Diet in Renal Disease Study (MDRD, 4-variable version) (18). The quantitative determination of total 25-hydroxyvitamin D (25(OH)D) levels was measured using the Access 25(OH) Vitamin D Total assay on the Access 2 Immunoassay System (Beckman Coulter, Inc., Brea, CA, USA, ref B24838), routinely used in our center. Total 25(OH)D levels represent the most reliable biomarker of vitamin D status (19). The liquid chromatography tandem-mass spectrometry (LC‐MS/MS) is considered to be a gold standard technique for measurement of vitamin D metabolites. However, the Access 25(OH) Vitamin D Total assay allows measurement of 25(OH)D serum levels with appropriate precision (**Supplementary Table 1**), has appropriate analytical values, and can be used in routine measurement of 25(OH)D (20, 21). According to the diagnostic threshold for serum 25(OH)D concentration approved for Poland, vitamin D concentration was interpreted as VDD when 25(OH)D concentration was ≤20 ng/ml, suboptimal between 20.1 and 30 ng/ml, and optimal between 30.1 and 50 ng/ml (22). For all comparative statistical analyses, suboptimal and optimal 25(OH)D concentrations were considered together as sufficient 25(OH)D levels (>20 ng/ml).

All patients included in the study underwent coronary angiography through a Philips Allura Xper DF20 X-ray system using a radial access and standard diagnostic catheters. All examinations were performed by the same certified cardiologist, who was blinded to the other results. The diagnosis of obstructive CAD was based on the detection of at least one stenosis ≥50% in any major epicardial artery.

### Statistical Analysis

Continuous data was presented as mean ± SD or median with interquartile range. Categorical variables were presented descriptively. A Shapiro–Wilk test was used to evaluate the normality of variables. The correlation between lipid profile and vitamin D concentration was checked using the Spearman correlation coefficient. The statistical significance of the Spearman correlation proves the existence of a monotonic dependence between the variables. In the case of comparing the values of a given numerical variable in two groups, the Mann-Whitney U test was used to assess statistical significance. The Fisher’s exact test was used to determine the relationship between VDD and clinical variables. Multivariate logistic regression analysis was performed to test the combined relationship between the deficiency of vitamin D and selected clinical parameters. Based on the Akaike information criterion (AIC), the best-fit model was selected. On the basis of the coefficients β, the value of their exponents: exp(β) was calculated, which means the unit odds ratio (OR). A p-value < 0.05 was considered to be significant for all statistical analyses. All statistical analyses were performed using the R software for statistical computing version 3.6.

## Results

### Participant Demographics

The study population included 48 patients (24 males and 24 females; mean age: 41.6 ± 8 years). At the presentation, the majority of patients (n = 32; 66.7%) were eligible for SPKT and the others for PTA according to transplant team decision. The baseline characteristics of the enrolled patients are illustrated in **Table 1**. The duration of diabetes in the study group was on average 26.5 ± 8.15 years, with more than 75% of patients (n = 37) with long-standing diabetes (over 20 years). The recommended goal of HbA1c level (<7%) was achieved by only 13 (27%) patients. There were 27 (56.2%) patients on HD with a mean duration of HD of 26.8 ± 18.1 months, and at least one in four patients had been undergoing this procedure for 3 years or more (n = 7; 25.92%). Atherosclerotic cardiovascular disease was diagnosed in 11 (23%) patients prior to the study entry (5 patients with stroke, 3 patients with peripheral arterial disease, and 3 patients with CAD). The hypertension and dyslipidemia were the most common cardiovascular risk factors in our cohort (n = 37; 77.1% and n = 30; 62.5%, respectively). The majority of patients presented with dyslipidemia were treated with statins (n = 26; 54%), while the remaining patients were only adopting dietary measures. The patients treated with statins reported the use of atorvastatin at the average dose of 12.69 mg/day. Obesity was found in only three participants (6.25%), and the largest group of patients presented with normal body weight (n = 34; 70.83%). The underweight was found in 2 (4.17%) patients and overweight in 9 (18.75%) patients. Only 11 (22.9%) subjects reported the use of vitamin D supplements during the last three months. Six of them were using cholecalciferol at the average dose of 1250 IU/day. The other patients reported the use of vitamin D or multivitamin supplements only occasionally. Therefore, the data on the mean dose of vitamin D in this subgroup were not available.

**Table 1:** Baseline characteristics of the study group divided into subgroups according to 25(OH)D concentrations.

Variable	Total (N = 48)	Vitamin D		p-value
		Deficiency (≤ 20 ng/ml) (N = 23)	Sufficient level (>20 ng/ml) (N = 25)	
Sex (females)	24 (50%)	12 (52.2%)	12 (48%)	1.000
Age [years]	42 (36.75–48)	43 (33.5–48)	42 (38–46)	0.992
Age of diagnosis of T1D [years]	14 (9–21)	15 (9–21.5)	13 (8–17)	0.332
Duration of T1D [years]	26 (21–33)	25 (19.5–30.5)	28 (24–34)	0.189
Duration of HD [months]	27 (14–36)	25.5 (12–31.5)	28 (24–36)	0.349
BMI [kg/m^2^]	23.06 (20.93–25.2)	23.59 (20.14–27.6)	22.86 (21.78–24.31)	0.845
Underweight	2 (4.17%)	1 (4.35%)	1 (4%)	
Normal weight	34 (70.83%)	15 (65.28%)	19 (76%)	
Overweight	9 (18.75%)	5 (21.74%)	4 (16%)	
Obesity	3 (6.25%)	2 (8.69%)	1 (4%)	
HbA1c [%]	7.54 (6.94–8.51)	7.75 (7.16–8.87)	7.53 (6.91–8.35)	0.516
eGFR [ml/min/1.73 m^2^]	12.5 (9.47–79.05)	10.5 (9.15–16.8)	25 (10.4–91)	**0.024**
25(OH)D [ng/ml]	20.19 (15.58–28.52)	15.28 (8.73–18.52)	28.33 (24.81–32.44)	Not applicable

Continuous variables are presented as median with interquartile range (IQR) and categorical variables as number and percentage (%). T1D, type 1 diabetes; HD, hemodialysis; BMI, body mass index; Underweight, BMI under 18.5 kg/m^2^; Normal weight, BMI 18.5–24.9 kg/m^2^; Overweight, BMI 25–29.9 kg/m^2^; Obesity, BMI ≥ 30 kg/m^2^; HbA1c, glycated hemoglobin; eGFR, estimated glomerular filtration rate calculated using the MDRD formula (see Materials and Methods); 25(OH)D, 25-hydroxyvitamin D.

Significant difference are marked in bold.

### Overall Characteristics of CAD in the Study Group

All patients were clinically asymptomatic and denied angina symptoms. Coronary angiography revealed that 17 (35% of the entire cohort) had CAD. Among patients with CAD, 5 out of 17 (29.4%) have single-vessel disease, 7 (41.2%) two vessel disease, and 5 (29.5%) multivessel disease. CAD treatment decisions were based on the results of the coronary angiography and invasive FFR (fractional flow reserve) or SPECT (single-photon emission computed tomography) according to the Heart Team decision. Among patients with CAD, 2 out of 17 (11.8%) were eligible for coronary artery bypass graft (CABG), 4 (23.5%) underwent percutaneous coronary intervention (PCI), 10 (58.8%) were qualified for optimal medical therapy, while 1 (5.9%) participant was not eligible for pancreas transplant due to multivessel, diffuse coronary lesions and the lack of technical possibilities for revascularization.

### Vitamin D Concentration and Its Correlations With CAD and Lipid Profile

The mean 25(OH)D concentration in the whole study group was 21.3 ± 9.48 ng/ml. The VDD (25(OH)D (concentration ≤ 20 ng/ml) was revealed in 23 (47.9%) patients, the suboptimal level of vitamin D (between 20.1 and 30 ng/ml) in 14 (29.2%) patients, and the optimal level (between 30.1 and 50 ng/ml) in only 11 (22.9%) patients.

A significant relationship between 25(OH)D concentration and CAD was proven by the Mann-Whitney U test. The median value of 25(OH)D concentration in patients with CAD was lower by 6.3 ng/ml than in patients without CAD (18.5 (11.6–21.5) *vs*. 24.8 (18.4–31.8) ng/ml, p = 0.018). It is worth noting that in both patients with advanced CAD qualified for CABG, the concentration of 25(OH)D was very low (10.51 ng/ml and 13.32 ng/ml) and the optimal concentration of 25(OH)D was reported only in one case in the CAD group (30.3 ng/ml).

In addition, we found a significant difference in the concentration of 25(OH)D depending on the status of arterial blood pressure. The median value of 25(OH)D concentration in patients with hypertension was significantly lower than in the group of patients with normal blood pressure [19.4 (13.3–25.8) *vs*. 29.1 (21.4–32.3) ng/ml, p = 0.026]. There were no significant relationships between 25(OH)D concentration and other variables (dyslipidemia, statin use, smoking habit, sex) (**Supplementary Table 2**).

Based on Spearman’s correlation matrix, we found only non-significant inverse correlation between 25(OH)D concentration and TC, LDL-C, TG, and non-HDL-C as well as non-significant positive correlation between concentration of 25(OH)D and HDL-C (**Table 2**). However, we did not prove any significant correlation between the concentration of 25(OH)D and lipid profile parameters. Furthermore, we did not confirm any significant association among 25(OH)D concentration and the other analyzed clinical and laboratory parameters (age, BMI, duration of T1D, duration of HD, HbA1c).

**Table 2:** Spearman correlation analysis testing the correlation between 25(OH)D concentration and selected laboratory and clinical parameters.

	TC	LDL-C	HDL-C	Non-HDL-C	TG
25(OH)D	r = -0.19	r = -0.17	r = 0.11	r = -0.23	r = -0.22
	p = 0.20	p = 0.25	p = 0.45	p = 0.11	p = 0.13
	**Age**	**BMI**	**Duration of T1D**	**Duration of HD**	**HbA1c**
25(OH)D	r = -0.12	r = -0.07	r = 0.12	r = -0.20	r = -0.26
	p = 0.40	p = 0.64	p = 0.40	p = 0.16	p = 0.08

r = Spearman correlation coefficient; 25(OH)D, 25-hydroxyvitamin D; TC, total cholesterol; LDL-C, low-density lipoprotein cholesterol; HDL-C, high-density lipoprotein cholesterol; non-HDL-C, non-high-density lipoprotein cholesterol; TG, triglycerides; BMI, body mass index; T1D, type 1 diabetes; HD, hemodialysis; HbA1c, glycated hemoglobin.

### Relationship Between VDD and CAD

**Figure 1:**
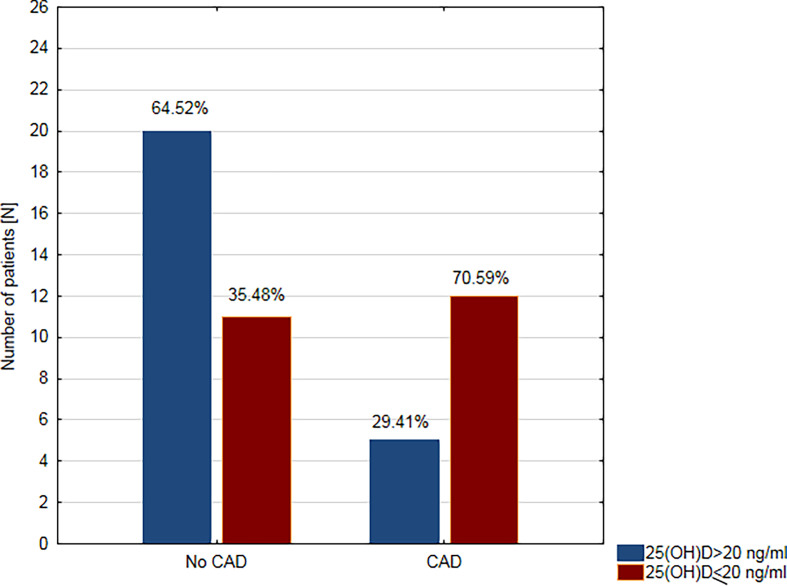
Prevalence of vitamin D deficiency in patients with coronary artery disease (CAD) and without coronary artery disease (no CAD).

In the next step, the study population was divided into two groups according to the concentration of 25(OH)D: deficiency (≤20 ng/ml) and sufficient level (>20 ng/ml). The deficiency of vitamin D was demonstrated in 23 (47.9%) patients, while the sufficient level was found in 25 (52.1%) participants. VDD was found in the majority of patients with CAD (n = 12; 70.59%), while there were only 11 (35.48%) subjects with VDD in the group without CAD (**Figure 1**).

**Figure 2:**
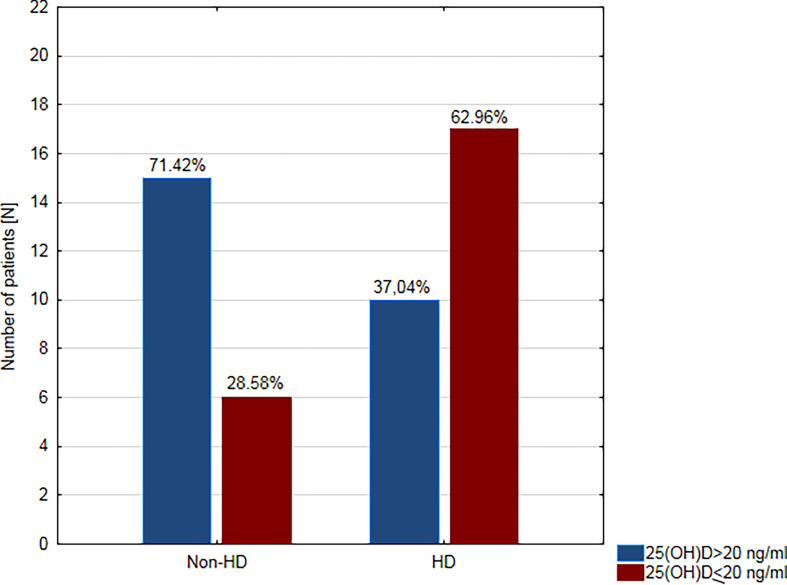
Prevalence of vitamin D deficiency in hemodialysis (HD) and non-dialysis patients (non-HD).

The relationship between deficient/sufficient level of vitamin D and CAD and the other clinical variables was tested using Fisher’s Exact Test (**Table 3**). There was a significant relationship between VDD and CAD (OR = 4.36; 95% confidence interval (CI): 1.22–15.64, p = 0.034. A patient’s odds of having CAD while having a sufficient level of vitamin D was 4.36 times lower than if the patient had VDD. Moreover, we found a significant relationship between VDD and hypertension (OR = 5.91; 95% CI: 1.12–31.20, p = 0.039) and HD (OR = 4.25; 95% CI: 1.25–14.5, p = 0.023). VDD was found in approximately two-thirds of hemodialysis patients (n = 17; 62.96%) and only in about one-third of non-dialysis patients (n = 10; 28.57%) (**Figure 2**). We did not prove any significant association between VDD and the prevalence of dyslipidemia, smoking habit, and the use of statins (**Table 3**).

**Table 3:** The relationships between vitamin D levels and the presence/absence of coronary artery disease, hemodialysis, main cardiovascular risk factors, and statins use.

Variable		Total (N = 48)	Vitamin D		OR (95% CI)	p-value
			Deficiency (≤20 ng/ml)	Sufficient level (>20 ng/ml)		
Hemodialysis	Yes	27 (56.2%)	17 (35.4%)	10 (20.8%)	4.25 (1.25- 14.5)	**0.023**
	No	21 (43.8%)	6 (12.5%)	15 (31.3%)		
Hypertension	Yes	37 (77.1%)	21 (43.8%)	16 (33.3%)	5.91 (1.12–31.20)	**0.039**
	No	11 (22.9%)	2 (4.2%)	9 (18.7%)		
Dyslipidemia	Yes	30 (62.5%)	17 (35.4%)	13 (27.1%)	2.62 (0.77–8.83)	0.145
	No	18 (37.5%)	6 (12.5%)	12 (25%)		
Statins use	Yes	26 (54.2%)	14 (29.2%)	12 (25%)	1.69 (0.53–5.31)	0.401
	No	22 (45.8%)	9 (18.7%)	13 (27.1%)		
Smoking habit	Yes	15 (31.2%)	9 (18.7%)	6 (12.5%)	2.03 (0.59–7.05)	0.353
	No	33 (68.8%)	14 (29.2%)	19 (39.6%)		
Coronary artery disease	Yes	17 (35.4%)	12 (25%)	5 (10.4%)	4.36 (1.22–15.64)	**0.034**
	No	31 (64.6%)	11 (22.9%)	20 (41.7%)		

Data are presented as number and percentage (%) of the entire cohort. OR, odds ratio; CI, confidence interval.

Significant difference are marked in bold.

We did not find any significant associations between VDD and lipid profile parameters: TC, LDL-C, HDL-C, non-HDL-C, TG (**Table 4**), as well as between VDD and age, duration of T1D, duration of HD, BMI, and HbA1c (**Table 1**). However, the median value of eGFR was significantly lower in vitamin D deficient participants than in participants with sufficient level of vitamin D (10.5 (9.15–16.8) *vs*. 25 (10.4–91) ml/min/1.73m^2^, p = 0.024) (**Table 1**).

**Table 4:** Lipid profile parameters in the entire cohort divided into subgroups according to 25(OH)D concentrations.

Variable	Total (N = 48)	Vitamin D		p-value
		Deficiency (≤20 ng/ml) (N = 23)	Sufficient level (>20 ng/ml) (N = 25)	
TC [mmol/L]	4.55 (3.88–5.3)	4.7 (4.05–5.7)	4.3 (3.8–5)	0.247
LDL-C [mmol/L]	2.4 (1.87–2.8)	2.6 (2.05–2.85)	2.2 (1.7–2.8)	0.283
HDL-C [mmol/L]	1.4 (1.2–1.6)	1.3 (1.2–1.55)	1.5 (1.3–1.6)	0.275
non-HDL-C [mmol/L]	3.15 (2.57–3.5)	3.2 (2.75–3.95)	3 (2.4–3.3)	0.137
TG [mmol/L]	1.5 (1.1–2)	1.5 (1.2–2.05)	1.3 (1.1–1.9)	0.138

Data are presented as median with interquartile range (IQR). TC, total cholesterol; LDL-C, low-density lipoprotein cholesterol; HDL-C, high-density lipoprotein cholesterol; non-HDL-C, non-high-density lipoprotein cholesterol; TG, triglycerides.

Multivariate logistic regression analysis was performed to test the combined interactions among vitamin D level (deficient/sufficient level) and selected clinical parameters. Initially, the model included all the variables that were significant in the univariate analysis (CAD, HD, hypertension, eGFR). Backward elimination process (with AIC-based comparisons) performed using step function excluded the hypertension, CAD, and eGFR from the model. Finally, in multivariate analysis, HD was the independent predictor of VDD in the study group (OR = 4.25; 95% CI: 1.25–14.5, p = 0.02) (**Table 5**). Having HD increases the risk of VDD by 4.25, with other variables unchanged.

**Table 5:** Univariate and multivariate logistic regression analyses performed to test the combined interactions between vitamin D levels and selected clinical parameters.

	Univariate logistic regression analysis			Multivariate logistic regression analysis		
	β (SD)	OR (95% CI)	p-value	β (SD)	OR (95% CI)	p-value
CAD	1.473 (0.651)	4.36 (1.22–15.64)	0.02	1.036 (0.703)	2.82 (0.71–11.18)	0.1
HD	1.447 (0.626)	4.25 (1.25–14.50)	0.02	1.447 (0.626)	4.25 (1.25–14.50)	**0.02**
HT	1.776 (0.849)	5.91 (1.12–31.20)	0.04	0.836 (1.011)	2.30 (0.32–16.74)	0.4
eGFR	-0.022 (0.009)	0.98 (0.961–0.996)	0.02	-0.008 (0.013)	0.99 (0.966–1.018)	0.5

The multivariate model included all the variables that were significant in the univariate analysis (coronary artery disease, hemodialysis, hypertension, eGFR). Based on the Akaike Information Criterion (AIC), the best-fit model was selected. On the basis of the coefficients β, the value of their exponents: exp(β) was calculated, which means the unit odds ratio. SD, standard deviation; OR, odds ratio; CI, confidence interval; CAD, coronary artery disease; HD, hemodialysis; HT, hypertension; eGFR, estimated glomerular filtration rate calculated using the MDRD formula (see Materials and Methods).

Significant difference are marked in bold.

## Discussion

To the best of our knowledge, this is the first study focusing on vitamin D levels and its correlation with CAD and lipid profile in pancreas transplant candidates. The study included all consecutive patients referred to our center for cardiological evaluation prior to pancreatic transplantation, without any bias in the selection of patients. This group includes middle-aged patients with long-standing T1D and multi-organ complications who continued their treatment, including vitamin D supplementation. The obtained results reflect the actual population of pancreas transplant recipients, so the conclusions can be applied in everyday clinical practice.

There are no studies specifically aimed at assessing VDD in pancreas transplant candidates with T1D. Therefore, our results may be compared to the general population and the overall diabetic population. In our study, the percentage of patients diagnosed with vitamin deficiency is higher when comparing our data to European data for the general population (48% *vs*. 40%) (7). The geographic location of Poland with low sunlight and insufficient exposure to ultraviolet light is probably the main cause of such finding. However, VDD in individuals with T1D is very common, even in a sun-rich environment (23). Moreover, the prevalence of VDD is higher in young patients with T1D compared to adolescents without diabetes (24–26). Referring to the Polish data of vitamin D status from 2016, it should be noted that in our group VDD occurs less frequently than in Polish adolescents with T1D (48% *vs*. 82%) (10). This is most likely due to the fact that almost a quarter of subjects of our study used vitamin D supplements.

Our study results demonstrate a high prevalence of CAD in T1D patients eligible for pancreas transplantation. CAD was found in 35% of clinically asymptomatic subjects. Comprehensive comparison with other studies is difficult due to the small number of previous studies addressing this issue. There are many reports on cardiovascular complications in the perioperative period, but unfortunately, there is little data on the prevalence of CAD in pancreatic transplant recipients. In addition, researchers use different criteria for the diagnosis of CAD. Michel et al. revealed CAD in 28 (47.5%) out of 59 pancreas recipients, but the diagnosis of CAD was based on the presence of even discrete stenosis of the coronary arteries (10–30% in the majority of patients) (27). Mangus et al. demonstrated that pre-transplant prevalence of CAD was 19% in pancreas transplant candidates with T1D (28), that is much lower than the frequency found in our study (35%). The study conducted by Mangus et al. was retrospective and the diagnosis of CAD was based on medical review; CAD was confirmed if the patient had ever been diagnosed with CAD. The mentioned authors point out that seriously ill patients who did not have a negative stress test were not subjected to transplantation and therefore were not included in the analysis (28). This may be a possible explanation for the underestimation of the prevalence of CAD in the aforementioned study.

The key finding of the present study is a significant correlation between vitamin D concentration and the prevalence of CAD. The concentration of 25(OH)D in patients with CAD was significantly lower than in patients without CAD. VDD was found in the majority of patients with CAD, and only in one-third of those without CAD. There was a significant correlation between VDD and CAD; a patient’s odds of having CAD while having a sufficient level of vitamin D was 4.36 times lower than if the patient had VDD. In this regard, the results of our analysis are consistent with numerous reports. A meta-analysis of 25 prospective studies conducted by Gholami et al. showed that low concentrations of vitamin D increase the relative risk of cardiovascular disease by 44% in the general population (29). According to the results presented by other researchers, VDD was associated not only with higher prevalence but also with the extent of CAD in both diabetic (type 2) and non-diabetic population (30–32). Moreover, vitamin D level was lower in patients with acute coronary syndrome than in control group and was the lowest in the subgroup of diabetic patients (33). VDD presents prognostic significance, as well. Karakas et al. demonstrated an inverse relationship between level of vitamin D and the risk of myocardial infarction during the 11 years of follow-up in the general population (34). Studies on the relationship of VDD with CAD in T1D patients are scarce, and their results are divergent. Young et al. demonstrated that VDD independently predicts the prevalence and progression of coronary artery calcification in adults with T1D (35). However, other researchers found no association between these factors (36, 37).

We demonstrated that eGFR was significantly lower in vitamin D deficient participants than in participants with sufficient level of vitamin D. These results are consistent with the results form Kim et al. who reported that VDD correlated with eGFR and worsening renal function from stage 3–5 (38). In addition, other researchers reported that VDD can be associated with diabetic nephropathy in both type 1 and type 2 diabetes (39, 40). Moreover, diabetic patients on hemodialysis have significantly lower levels of vitamin D than patients without diabetes (41). On the other hand, Diaz et al. reported that diabetic patients with nephropathy present significantly higher prevalence of VDD than patients without nephropathy (42). Therefore, our study results are consistent with the results from other researchers suggesting a link between diabetes, diabetic nephropathy, and VDD. Pancreas transplant candidates with T1D are at increased risk of VDD. Therefore, measurement of serum 25(OH)D levels plays a pivotal role in the prompt diagnosis and treatment of VDD in this group of patients. In this regard, Felício et al. showed that high-dose vitamin D3 supplementation (4,000 or 10,000 IU/day based on baseline vitamin D levels) for 12 weeks led to an improvement of the diabetic kidney disease stage in 62% of patients with T1D (43).

To explain the relationship between the concentration of vitamin D and CAD, we examined the association between vitamin D and the lipid profile, as well. Surprisingly, we did not find any significant relationship between the concentration of vitamin D and dyslipidemia, smoking and use of statins. We only found a non-significant inverse correlation between 25(OH)D concentration and TC, LDL-C, TG, and non-HDL-C as well as non-significant positive correlation between 25(OH)D concentration and HDL-C. Our findings are in contrast with most previous studies, which confirmed the beneficial effects of higher levels of vitamin D on lipid profile. It is worth noting that the results of research on this issue are inconsistent, as well. Jorde et al. demonstrated that high levels of vitamin D are associated with low TG and with high LDL-C, HDL-C, and TC in the general population (44). However, when researchers conducted an analysis on the subgroup of subjects who reported low consumption of food and supplements containing vitamin D, only the association between 25(OH)D and HDL cholesterol was significant. The authors conclude that the relationship between 25(OH)D and the lipid profile (with the exception of HDL cholesterol) can be explained by known confounding factors (e.g. diet) and does not reflect the causal relationship. Yarparvar et al. revealed that the HDL level was lower in the group with 25(OH)D levels <25 ng/ml compared to the group with 25(OH)D levels ≥25 ng/ml. A significant positive association was found between serum levels of vitamin D and HDL (45). The mentioned authors found a significant negative association between vitamin D level and both serum tumor necrosis factor receptor-2 (TNFR-2) and high-sensitive C-reactive protein (hsCRP) in that study. Moreover, in patients with 25(OH)D levels ≥25 ng/ml and negative hsCRP, the levels of interleukin 10 (IL-10) were higher than in the other groups. Based on the obtained data, Yarparvar et al. conclude that serum vitamin D could affect an inflammation status. Zambrana-Calví et al. found that children with T1D and VDD had higher TG levels than the patients with T1D and sufficient vitamin D levels (25(OH)D concentrations above 20 ng/ml) (16). The main explanation for our results may be that the majority of our participants used statins, which may have affected the relationship between vitamin D levels and the lipid profile. On the other hand, our findings are consistent with the results from the study conducted by Hafez et al, who did not show any significant association between vitamin D status and lipid profile in children with T1D (15). Overall, the association between VDD and the increased incidence of CAD in our study is likely due to non-lipid effects.

There are many other potential mechanisms that may explain the relationship between vitamin D and CAD. It is worth considering that vitamin D acts *via* its active form - 1,25-dihydroxyvitamin D [1,25(OH)2D] through nuclear vitamin D receptor (VDR). The VDR is located in most tissues including vascular smooth muscle, endothelial cells, cardiomyocytes, platelets, macrophages, and other immune cells (46–48). Thus, in addition to calcium metabolism, vitamin D is involved in the regulation of many systemic processes. Vitamin D has been shown to inhibit vascular smooth muscle cell proliferation, suppress vascular calcification, and modulate inflammatory cytokines (49). VDD leads to an imbalance of endothelial inflammatory factors by increasing the level of pro-inflammatory cytokines and reducing anti-inflammatory ones (50). Vitamin D inhibits the expression of adhesion molecules that leads to anti-atherosclerotic effects (51). *In vitro* studies demonstrated that 1α,25-(OH)(2)D(3) inhibits vascular cell adhesion molecule-1 (VCAM-1) and intercellular adhesion molecule-1 (ICAM-1) expression, interleukin-8 (IL-8) production, monocyte chemoattractant protein-1 (MCP-1) secretion, and monocyte adhesion in human endothelial cells (52, 53). Another potential explanation for the association between VDD and CAD is the link with matrix metalloproteinases (MMPs). Increased activity of MMP mediates the degradation of extracellular matrix proteins leading to atherosclerotic changes in the vascular wall (54). Timms at al. revealed an association between vitamin D insufficiency and increased circulating levels of MMP-2 and MMP-9 and demonstrated that vitamin D supplementation resulted in a significant reduction in MMP-9 (55). Furthermore, there is a strong evidence to support the role of VDD in the activation of renin–angiotensin system (RAS) that leads to hypertension (56). It is worth noting that in our study 25(OH)D concentration shows a significant inverse correlation with hypertension; the median value of 25(OH)D concentration in patients with hypertension was significantly lower than in the group of patients with normal blood pressure. A link between VDD, RAS activation, hypertension, and CAD could be another possible explanation for our study results.

In addition to the potential protective role of vitamin D in T1D patients in the pre-transplant period, the role of vitamin D in pancreas recipients in the post-transplant period should also be considered. Below we will discuss the importance of diagnosing and correcting the VDD in pancreas transplant candidates also in light of the anti-inflammatory and immunomodulatory properties of vitamin D. Indeed, pre-clinical studies have shown that vitamin D may have the potential to promote graft survival and prevent recurrence of autoimmunity, as well as allograft rejection in animal models of syngeneic and allogeneic islet transplantation (57). Moreover, observational studies have shown that VDD is also highly prevalent after solid organ transplantation (e.g., lung, liver, or kidney transplantation) (58, 59). In addition, VDD appears to be an independent risk factor for post-transplant diabetes mellitus (PTDM) after kidney transplantation (60).

Our findings have important implications for the clinical management of pancreas transplant candidates with T1D and for the potential prevention of complications resulting from pancreas transplantation. T1D patients eligible for pancreas transplantation constitute a very specific group of patients. First, a multitude of risk factors increase the likelihood of perioperative cardiovascular complications in this group of individuals. Second, pancreas transplantation is a valid treatment option for T1D patients with imminent or established end-stage renal disease who have had or plan to have a kidney transplant (61). Therefore, we should make every effort to identify and eliminate all potential risk factors to minimize perioperative risks and provide pancreas transplantation for all subjects who may benefit from it. VDD may represent an additional, modifiable cardiovascular risk factor among pancreas transplant candidates with T1D. The measurement of 25(OH)D concentration should be considered in all type 1 diabetic patients eligible for pancreas transplantation. The supplementation of vitamin D seems to be a potential, inexpensive therapeutic option that could help to reduce the risk of CAD and thus improve periprocedural prognosis in this group of patients.

Our study has, however, some limitations. The first limitation is due to the cross-sectional nature of the study. The analysis demonstrates only a single timepoint assessment of vitamin D status. As an observational study, it has exploratory purposes and no causal relationships can be established. Second, the sample size in the present study is relatively small. The main reason is that in Poland a total of about 30 PTA/SPKT procedures are performed annually (62); therefore, the number of patients eligible for pancreas transplantation is proportionally limited. Nevertheless, apart from the low number of subjects, the group we studied is fully representative from a practical point of view. The third limitation is the use of statins. Statins improved lipid parameters and thus may potentially interfere with assessment of the correlation between vitamin D status and lipid profile. However, the study was aimed to test the actual population of pancreas transplant candidates; therefore, the authors decided not to select the patients involved.

## Conclusions

VDD is highly prevalent in pancreas transplant candidates with T1D. There is a significant relationship between VDD and a higher prevalence of CAD. The lack of any significant association between serum vitamin D and lipid profile suggests that the relationship between vitamin D and CAD results from other causes. Further research is therefore needed to confirm the existence of a cause-effect relationship between VDD and CAD.

## Data Availability Statement

The raw data supporting the conclusions of this article will be made available by the authors, without undue reservation.

## Ethics Statement

The studies involving human participants were reviewed and approved by Bioethics Committee of the Medical University of Warsaw, Poland. The patients/participants provided their written informed consent to participate in this study.

## Author Contributions

MB-L conceived and designed the study, collected and analyzed the data, wrote the manuscript, and edited the final version. PK and RR created the database and analyzed the data. WL contributed to data collection and to a critical revision of the manuscript. AM contributed to a critical revision of the manuscript for important intellectual content. All authors contributed to the article and approved the submitted version.

## Funding

Medical University of Warsaw, Poland (publication fee). 

## Conflict of Interest

The authors declare that the research was conducted in the absence of any commercial or financial relationships that could be construed as a potential conflict of interest.

## Publisher’s Note

All claims expressed in this article are solely those of the authors and do not necessarily represent those of their affiliated organizations, or those of the publisher, the editors and the reviewers. Any product that may be evaluated in this article, or claim that may be made by its manufacturer, is not guaranteed or endorsed by the publisher.
