# Supplementary material for: Vitamin D Deficiency as a Predictor of a High Prevalence of Coronary Artery Disease in Pancreas Transplant Candidates With Type 1 Diabetes
Source: Front Endocrinol (Lausanne). 2021 Aug 11;12:714728. doi: 10.3389/fendo.2021.714728 (PMC8385141; doi:10.3389/fendo.2021.714728)
Supplement: Supplementary file 1 [file Table_1.docx]

Supplementary Table 1. Characteristics of the Access 25(OH) Vitamin D Total assay on the Access 2 Immunoassay System according to data provided by manufacturer. 25(OH) vitamin D2, [ergocalciferol](https://en.wikipedia.org/wiki/Ergocalciferol); 25(OH) Vitamin D3, cholecalciferol; CV, coefficient of variation.

| **Parameter** | **Characteristics** |
| --- | --- |
| Reportable Measuring Range | 2.0 – 167 ng/mL |
| Cross Reactivity | 25(OH) vitamin D2 100%  25(OH) vitamin D3 100% |
| Precision | Total imprecision ≤ 10.0% CV at concentrations > 15.0 ng/mL  Total Standard Deviation (SD) ≤ 1.5 ng/mL at concentrations ≤ 15.0 ng/mL |
