# Supplementary material for: Vitamin D Deficiency as a Predictor of a High Prevalence of Coronary Artery Disease in Pancreas Transplant Candidates With Type 1 Diabetes
Source: Front Endocrinol (Lausanne). 2021 Aug 11;12:714728. doi: 10.3389/fendo.2021.714728 (PMC8385141; doi:10.3389/fendo.2021.714728)
Supplement: Supplementary file 2 [file Table_2.docx]

Supplementary Table 2. The comparison of 25(OH)D concentrations in the study group according to presence/absence of coronary artery disease and selected clinical factors. Data are presented as median with interquartile range (IQR).

| **Parameter** | **Variable** | | **p-value** |
| --- | --- | --- | --- |
| 25(OH)D [ng/ml] | Coronary artery disease | |  |
|  | Yes (N=17) | No (N=31) |  |
|  | 18.47 (11.63 - 21.49) | 24.81 (18.38 - 31.84) | **0.018** |
|  | Hypertension | |  |
|  | Yes (N=37) | No (N=11) |  |
|  | 19.44 (13.32 - 25.76) | 29.1 (21.38 - 32.31) | **0.026** |
|  | Dyslipidemia | |  |
|  | Yes (N=30) | No (N=18) |  |
|  | 19.32 (13.81 - 25.89) | 24.06 (19.11 - 31.62) | 0.123 |
|  | Statins use | |  |
|  | Yes (N=26) | No (N=22) |  |
|  | 19.7 (13.81 - 25.89) | 22.98 (18.1 - 31.62) | 0.191 |
|  | Smoking habit | |  |
|  | Yes (N=15) | No (N=33) |  |
|  | 18.56 (12.48 - 25.05) | 21.49 (17.34 - 30.26) | 0.251 |
|  | Sex | |  |
|  | Females (N=24) | Males (N=24) |  |
|  | 20.13 (17.38 – 31.37) | 20.8 (11.67 – 27.44) | 0.574 |
